# Transcriptome Analysis of the Hippocampus in Novel Rat Model of Febrile Seizures

**DOI:** 10.1371/journal.pone.0095237

**Published:** 2014-04-15

**Authors:** Zhongcheng Wang, Yuanteng Fan, Jian Xu, Liang Li, Duanhe Heng, Song Han, Jun Yin, Biwen Peng, Wanhong Liu, Xiaohua He

**Affiliations:** 1 Department of Pathophysiology, School of Basic Medical Sciences, Wuhan University, Wuhan, China; 2 Hubei Provincial Key Laboratory of Developmentally Originated Disease, School of Basic Medical Sciences, Wuhan University, Wuhan, China; 3 Hubei Province Key Laboratory of Allergy and Immunology, School of Basic Medical Sciences, Wuhan University, Wuhan, China; University of North Carolina School of Medicine, United States of America

## Abstract

Febrile seizures (FS) are the most common type of convulsive events in infants and young children, but the precise underlying genetic mechanism remains to be explored. To investigate the underlying pathogenic factors in FS and subsequent epilepsy, alterations in gene expression between the two new strains of rats (hyperthermia-prone [HP] vs hyperthermia-resistant [HR]), were investigated by using the Whole Rat Genome Oligo Microarray. This process identified 1,140 differentially expressed genes (DEGs; 602 upregulated and 538 downregulated), which were analyzed to determine significant Gene Ontology (GO) categories, signaling pathways and gene networks. Based on the GO analyses, the modified genes are closely related to various FS pathogenesis factors, including immune and inflammatory responses and ion transport. Certain DEGs identified have not been previously examined in relation to FS pathogenesis. Among these genes is *dipeptidyl peptidase 4* (*DPP4*), a gene closely linked to *interleukin 6* (*IL-6*), which played a key role in the gene network analysis. Furthermore, sitagliptin, a DPP4 inhibitor significantly decreased epileptic discharge in rats, observed via electroencephalogram, suggesting an important role for DPP4 in FS. The effectiveness of sitagliptin in reducing seizure activity may occur through a mechanism that stabilizes cellular Ca^2+^ homeostasis. In addition, DPP4 expression may be regulated by DNA methylation. The hippocampal gene expression profiles in novel rat models of FS provides a large database of candidate genes and pathways, which will be useful for researchers interested in disorders of neuronal excitability.

## Introduction

Febrile seizures (FS) are the most common seizure disorders in children, occurring in 2–5% of children before age 5 years [1], [2]. Retrospective studies with epilepsy showed that 10–15% of these patients had previous FS [3], [4]. In prospective follow-up studies of large cohorts of children with FS, afebrile seizures occur in 2–7%, which is up to ten times the prevalence in the general population [5]. FS are complex and heterogeneous, and genetic factors contribute significantly to the etiology of FS [6]. This is supported by the findings in twin and family studies which demonstrated an important genetic component in the etiology of FS [7], [8]. Eleven genetic loci (FEB1-11) have been mapped in the Online Mendelian Inheritance in Man (OMIM) database (http://www.omim.org/). In these genetic loci, some susceptibility genes (*Scn1a, Scn9a, IL-1β, IL-10, Gpr98* and *Cap6*) have been confirmed[6], [9]–[12], but not in other six other genetic loci FEB1, 2, 5, 7, 9 and 10. The precise underlying pathogenic factor is still unclear which has hampered many aspects of the study of FS.

Genetic animal models, particularly those with controlled genetic and epigenetic backgrounds, are useful tools for investigating the pathogenesis of a disorder without interferences from many confounding factors [13], [14]. Hyperthermia influences neuronal protein expression and cell properties by manipulating gene transcription and epigenetic modification[15]–[17]. Prolonged experimental FS have been found to enhance hippocampal network excitability and promote epilepsy, which may result from broad modifications of the gene-expression programs of numerous molecules that govern neuronal excitability and network response [18], [19]. We previously developed two new strains of FS rats by using an established model of hyperthermia-induced seizures combined with a selective breeding process, which were named Hyperthermia-Prone (HP, lower seizure-threshold) and Hyperthermia-Resistant (HR, higher seizure-threshold). The two strains of rats were housed under standard laboratory conditions and hyperthermia-induced seizures were produced using hot water bath immersion. The most sensitive rats from the HP group and the most resistant rats from the HR group were used as breeders to create the next generation. This process resulted in a heritable significant difference in seizure sensitivity and subsequent epilepsy between the two groups of rats [20]. As a result of the simple external interference in the modeling process, these two strains of rats provide a novel tool to study the underlying pathogenesis induced by hyperthermia in FS.

To identify genetic factors that contribute to the FS, especially in hippocampus, a gene microarray screen was performed to compare the hippocampus of the HP and HR strains. Furthermore, using a range of data mining and information annotation approaches, we were able to identify many candidate genes that were significantly differentially expressed between the two groups. These identified candidate genes might shed some light in elucidating the pathogenic factors in FS and the subsequent development of epilepsy.

## Materials and Methods

### Ethics Statement

All protocols complied with the recommendations in the Guide for the Care and Use of the Animal Biosafety Level 3 (ABSL-3) Laboratory and were approved by the Animal Ethics Committee of the Wuhan University (Permit Number: SCXK 2008-0004). All surgery was performed under chloral hydrate anesthesia, and all efforts were made to minimize suffering.

### Seizures Induction and Brain Sampling

Sprague-Dawley rats were obtained from the ABSL-3 Laboratory. Unless noted otherwise, 21 days old HP and HR rats at the beginning of the experiments were used. Rats were housed individually on a 12-h light-dark cycle with *ad libitum* access to food and water. Seizure induction and selective breeding procedure of HP and HR rat strains were carried out as previously described [20]. Hyperthermia-induced seizures were produced using hot water bath. The animals were placed in a temperature-controlled water bath and were immediately removed from the water when seizures were induced.

After the finish of the whole seizure-induction process, rats were anesthetized with 10% chloraldurate (3 ml/kg). The brains were quickly removed and placed in ice-cold, and the hippocampi were dissected and placed into liquid nitrogen, and transferred into a −80°C low-temperature refrigerator for storage and use.

### RNA Extraction and Microarray Hybridization

Three rats were selected randomly from HP group and HR group, respectively. Whose hippocampus were collected under RNase-free conditions immediately after sacrificing. Total RNA was isolated using the Trizol reagent (Invitrogen, CA) and purified with an RNeasy column (Qiagen, Germany). The RNA purity and concentration were confirmed by Nanodrop spectrophotometer (ThermoFisher, USA). The assessment of RNA integrity was identified with an Agilent 2100 Bio analyzer (Agilent Technologies, CA, USA). Samples were purified using a Qiagen RNeasy Kit (Qiagen, Germany). Microarray analysis was performed at CapitalBio Corporation (SBC, China) using Whole Rat Genome Oligo nucleotide 4×44 k Microarrays (Agilent, CA, USA). The quality of cyanine-labeled cRNA samples, including yield, concentration, amplification efficiency and abundance of cyanine fluorophore, was determined by an ND-1000 spectrophotometer (Nanodrop, USA) at A260 and A280. Once the concentration had been determined, cyanine-labeled cRNA fragmentation and microarray slide hybridization followed (Agilent Technologies). Following hybridization, the microarray slides were scanned using an Agilent microarray scanner G2565BA. Raw expression data were normalized using robust multiarray averaging with quantile normalization. The information produced by the scanner was loaded into the image analysis program Feature Extraction version 9.5 to establish standard data for statistical analysis, and all microarray slides were checked for background evenness.

### Differentially Expressed Genes (DEGs), Gene Ontology (GO) and Pathway Analysis

LIMMA (Linear Models for Microarray Data) and empirical Bayes methods were used to further investigation of the DEGs between the two groups. DEGs were considered significant if both the P value<0.05 and the fold change (FC)>1.5.

The GO annotations of the DEGs were downloaded from the GO project (http://www.geneontology.org) and NCBI (http://www.ncbi.nlm.nih.gov). The “elim Fisher” algorithm described by Alexa *et al* was used for the GO enrichment test [21]. GO categories with a *P* value<0.01 were reported.

The pathway analysis was obtained from the the Kyoto Encyclopedia of Genes and Genomes (KEGG) database (http://www.genome.jp/kegg). A Fisher exact test was used to find significant enrichment for pathways. Pathway categories with a *P*<0.05 were reported.

### Gene Network Analysis

Functional association networks were constructed using the GeneSpring GX software (version 11.0, Agilent Technologies, USA). The annotation data presented in this analysis were obtained from NCBI’s Gene Expression Omnibus (GSE23165).

All DEGs were selected to construct a co-expression network. The network edges were specified to feature Pearson correlation coefficients above 0.99 to ensure strong gene co-expression relationships. Degree description of the number of single gene that regulate other genes. Difdegree describe the difference in value between the HP and HR groups. A k-core of a gene co-expression network stands for the ability of co-expression.

### Quantitative Real-time PCR (qRT-PCR) Analysis

To confirm the microarray results, ten representative genes were confirmed by qRT-PCR, as described [22]. Six rats (HP n = 3 and HR n = 3) were selected randomly from each group. After hippocampus isolation and RNA extraction, briefly cDNA was prepared using the RevertAid™ First Strand cDNA Synthesis Kit (ThermoFisher, USA). By employing the Real Master mix (SYBR Green) Kit (Tiangen, China) and a LightCycler (Roche Diagnostics, Germany), qRT-PCR was performed following the manufacturer’s protocols. Single PCR products were further verified by melting curve analysis. Note that rat β-actin was always amplified in parallel with the representative genes. The relative expression ratio was determined by the formula 2^−ΔΔct^ method.

### Surgery and Electroencephalogram (EEG) Recording

SD rats (21 days old, n = 12) were surgically implanted with an injection guide cannula and recording electrodes using stereotaxic guidance under deep chloral hydrate anesthesia, as previously described [20]. Two electrodes were implanted bilaterally into the dorsal hippocampus (from the posterior fontanelle: nose bar 0; anteroposterior 2.5 mm, lateral 2.5 mm and 2.5 mm below dura mater). At the same time, a guide cannula was positioned unilaterally below the dura mater to enable the intracerebroventricular (icv) injection of drugs (from the bregma: nose bar 0;anteroposterior 1.5 mm, lateral 1.5 mm and 3.5 mm below the dura mater). EEGs recordings were obtained in freely moving rats 1 week after surgery. The baseline EEG for each rat was stable and reproducible after the drug injection or before exposure to hyperthermia. A first recording period (0–5 min) was used to assess the normal baseline before drug injection. Subsequently, the icv injection of drugs (n = 6) or vehicle (n = 6) was performed using a microsyringe and rats were allowed to rest for 20 min after injection. Another recording period (25–30 min) was used to assess the baseline before exposure to hyperthermia, and then EEG activity was measured continuously throughout the entire process including 4 min of exposure to warm water at a temperature of 44°C and an observation period. EEG recording were stopped after hyperthermia exposure and monitored until we observed a 10 min EEG trace that was similar to baseline. All EEG power data were recorded and analyzed using the RM6240 physiological signal acquisition and processing system (Chengdu instrument, China).

### Cell Culture and Intracellular Ca^2+^ Detection

The rat glioma C6 cell line (ATCC, USA) was grown in Dulbecco’s modified eagle medium (GIBCO, USA) supplemented with 15% heat-inactivated fetal calf serum, 50 U/ml penicillin and 50 µg/ml streptomycin. The culture dishes were kept at 37°C in a humidified atmosphere (5% CO_2_).

The fluorescence ratio of the Ca^2+^ indicator dye Fura-3-acetoxymethyl (AM) ester (Beyotime, China) was used to quantify Ca^2+^ concentrations in the C6 cells. The cells were incubated for 60 min in the presence of 2 µM Fura-3 AM, washed twice with Krebs-Hepes buffer and allowed to incubate with sitagliptin (100 µM) or glutamate (60 µM) for 20 min before imaging. The [Ca^2+^]i values are presented in terms of relative fluorescence intensity, and the fluorescence ratio (RFU) of [Ca^2+^]i was determined using the VICTOR ×5 Multilabel Plate Reader (PerkinElmer, USA). Fluo-3 was excited at a wavelength of 488 nm and fluorescence was measured at a wavelength of 515 nm. Values for each group represent the total [Ca^2+^]i of 3.5×10^4^ C6 cells.

### Western Blot

After decapitation, the freshly isolated hippocampal tissues were collected and protein content was determined by BCA Protein Quantitation Kit (Thermofisher, USA). The protein amount loaded per lane was 10 µg. After separation, the proteins were transferred to nitrocellulose paper and unspecific protein binding sites were blocked with 5% skim milk. Tublin was used as loading control for DPP4, The blots were incubated overnight with the primary antibodies, antibodies against DPP4 and Tublin were purchased from Abcam (1∶2000). Followed 2 h incubation with horseradish peroxidase-conjugated secondary antibody purchased from Proteinlab (1∶10,000). Immunoreactivity was visualized using the ECL detection system (Thermofisher, USA). Data were analyzed by Quantity one software.

### Bisulfite Sequencing

After decapitation, genomic DNA was extract from freshly isolated hippocampal tissues using DNA extraction kit (Yuanpinghao Bio, China) according to the manufacturer’s instructions and then modified with sodium bisulfite using EpiTect Bisulfite kit (Qiagen, Germany). DNA was then PCR amplified using the *dipeptidyl peptidase 4* (*DPP4*) CpG island 1 (−229/+94) primer pair forward: 5′-AGGGA GGAAT AGTTA AATTT TAATG TGTA-3′ and reverse: 5′-AAAAA ATCTA TCAAA ACACC ACCAC-3′ or CpG island 2 (+221/+325) primer pair forward: 5′-TGGAG GTAAT TAGGA GTTGG TAATA G-3′ and reverse: 5′-AAACA CTAAA TTTTA ACCCC AAAAC-3′. The PCR products were subcloned into pMD18-T simple vector (Takara, Japan) and sequencing was accomplished by Sangon Company (Shanghai, China).

### Statistics

Unless noted otherwise, statistical significance of differences between groups was assessed using Students t tests. Throughout the text, summary data were presented as mean ± SEM.

## Results

### Sample Clustering

**Figure 1:**
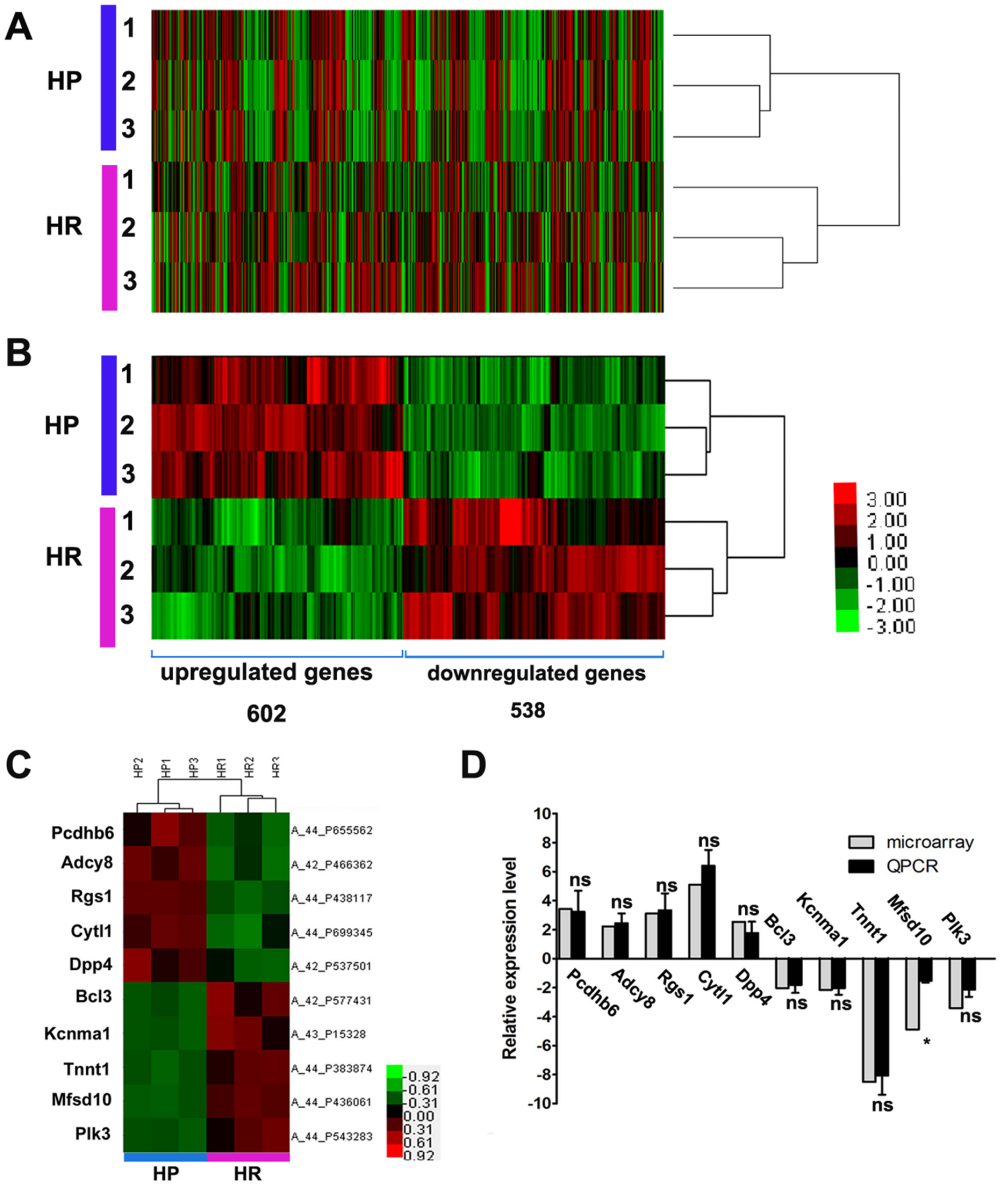
Hierarchical clustering analysis and qRT-PCR verification. (A) Hierarchical clustering analysis of all experimental samples. The reliable probes Horizontal stripes represent genes while columns show experimental samples (HP n = 3 and HR n = 3). Relationships were represented by the tree lines at the right side of the heat map (short line, close relation; long line, distant relation). (B) Heat map of the differentially expressed genes (DEGs) between the HP and HR groups. 1140 genes (*P*<0.05, FC>1.5) were identified from the hierarchical cluster, including 602 upregulated genes and 538 downregulated genes. Gene expression was shown in the heatmap as up-regulated (red color), down-regulated (green color) and no change (black color). (C) Hierarchical clustering analysis of 10 DEGs (*P*<0.05, FC>2). The genes (left) and probes (right) were shown in the heatmap. Gene expression profile was shown in the heatmap as up-regulated (red color), down-regulated (green color) and no change (black color). (D) The verification results of qRT-PCR. *Adcy8, Cytl1, Pcdhb6, Rgs1, Dpp4*, were up-regulated, *Bcl3, Kcnma1, Tnnt1, Mfsd10, Plk3* were down-regulated in the HP group compared with the HR group. Fold change was calculated based on the mean intensity value from 3 rats by using the comparative Ct method and normalized to the housekeeping gene *β-actin*, *P<0.05, ns: no significant difference.

Hierarchical sample classification was applied to the expression matrix, each data set generally separated the HP samples (n = 3) from the HR samples (n = 3) (Fig. 1A). All six samples were categorized into two main distinct clusters, corresponding to our expected groups. It was identified that 1140 genes (*P*<0.05, FC>1.5) were significantly different in the HP compared to the HR groups (Fig. 1B, Table S1), of which 602 or 538 genes were upregulated or downregulated, respectively. Notably, some known susceptibility genes of FS (*sodium channel, voltage-gated, type IX, alpha subunit [Scn9a]* and *interleukin-1 beta [IL-1β]*) were also identified in the DEGs.

To confirm the results obtained from the microarray, 10 selected DEGs (5 upregulated and 5 downregulated) were determined using qRT-PCR, based on their involvement in different functional groups and/or pathways. Our results suggested that majority of the investigated genes had congruent results between the microarray and qRT-PCR assays (Fig. 1C–D). Primers for the 10 genes were summarized in Table S2.

### GO and Pathway Analysis

**Figure 2:**
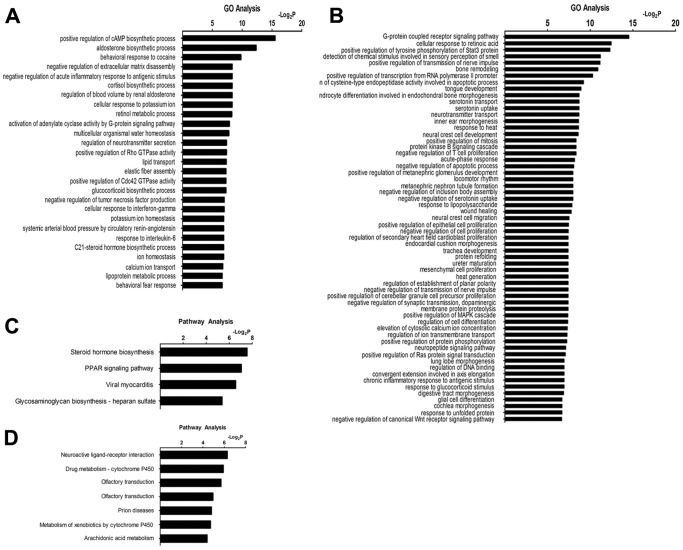
Gene ontology (GO) and pathway analysis. (A) The significant GO category for up-regulated genes between the HP and HR groups. (B) The significant GO category for down-regulated genes between the HP and HR groups. The vertical axis was the GO terms, and the horizontal axis is the enrichment of GO. (C)The significant pathway for up-regulated genes between the HP and HR groups. (D) The significant pathway for down-regulated genes between the HP and HR groups. The vertical axis was the pathway category, and the horizontal axis was the enrichment of pathways. P value<0.01 was used as a threshold to select significant GO categories, while P value <0.05 were used as thresholds to select significant KEGG pathways. -Log_2_P was the base 2 logarithm of the P value.

The significant GO categories were designated as those with a P value <0.01. The GO categories for genes that significantly differentially expressed between the HP and HR groups were associated with positive regulation of neurotransmitter secretion, negative regulation of synaptic transmission, and negative regulation of acute inflammatory response to antigenic stimulus (Fig. 2A–B). A portion of these GO terms were associated with inflammation (e.g. *interferon-gamma [IFN-γ]* and *interleukin-6 [IL-6]*). All significant GO terms of DEGs and related data were summarized in Table S3.

The significant KEGG pathways were designated as those with P value <0.05. The significant pathways of upregulated genes mainly included the steroid hormone biosynthesis pathway and PPAR signaling pathway (Fig. 2C). The significant pathways of downregulated genes mainly included neuroactive ligand-receptor interaction pathway and drug metabolism-cytochrome P450 pathway (Fig. 2D). The DEGs involved in significant pathways were listed in Table S4.

### Gene Network Analysis

**Figure 3:**
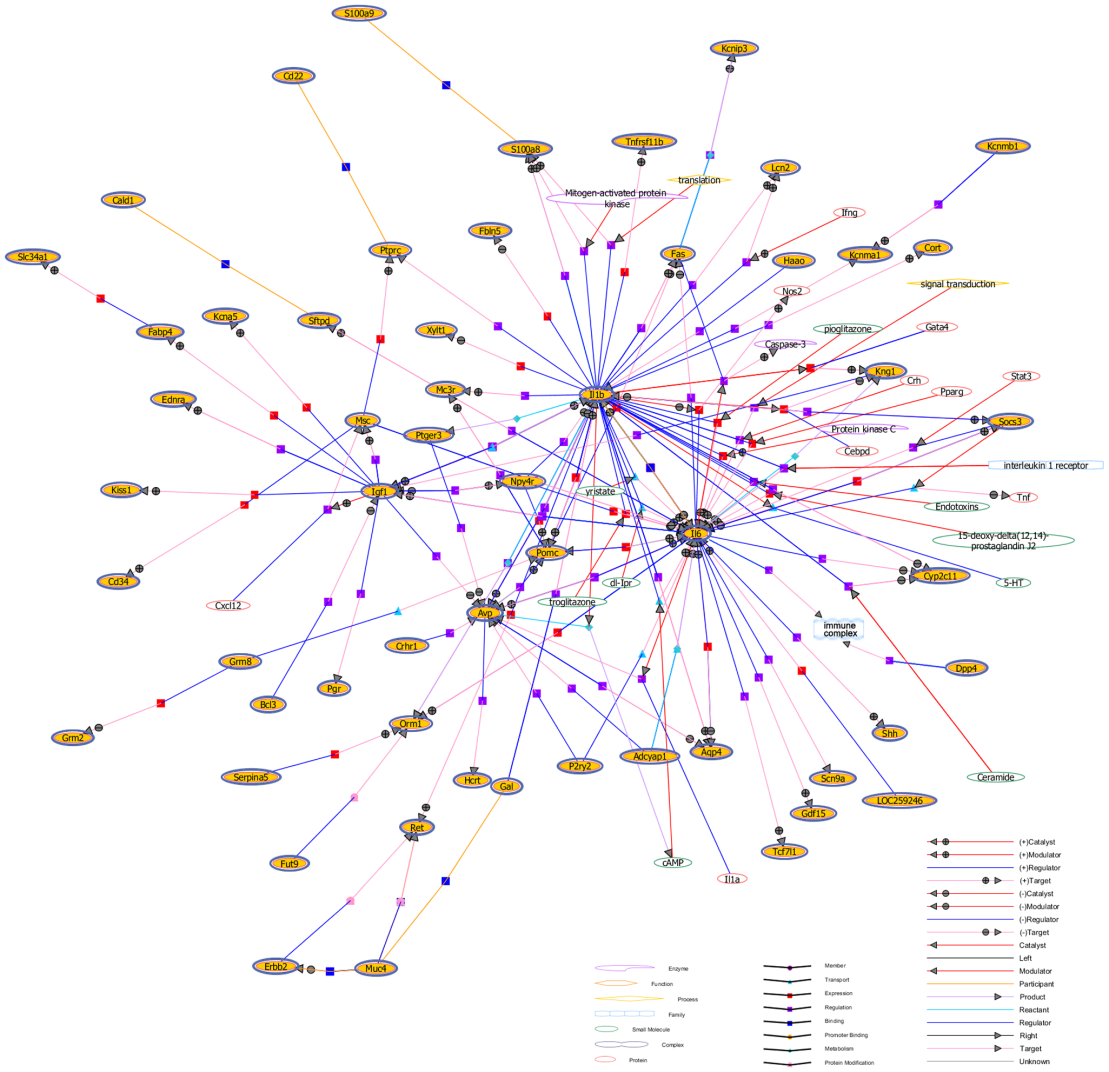
Prediction of DEGs relevance network. Genes were represented as yellow elliptic. The yellow oval-shaped pattern surrounded by a blue circle represents the protein available in the database that has been reported to interact with other proteins. Other uncolored nodal genes are directly or indirectly associated with the DEGs.

Genes do not function in isolation, but rather as interacting partners in complex molecular networks that control biological processes. To further study the relationships between DEGs, GeneSpring software were used to construct gene networks for direct and/or indirect contact among these genes, with integrative mining defining a molecular network depicting the generation of FS. In the current study, it was detected that *IL-1β* and *IL-6* were in important positions in the constructed network map; whereas other DEGs in the network were directly or indirectly associated with the *IL-1β* or *IL-6* (Fig. 3). Thus these data further suggests that immune response may be involved in the molecular network during the development of FS.

To identify the gene (s) play pivotal roles in the HP and HR groups, we also constructed a gene co-expression network that predicts the relationship among the DEGs. In this network, we applied the notion of k-core value to predict gene function similarity. Thirty-eight DEGs with higher degrees occupied more central positions within a large-scale gene network than other DEGs (Table S5), including ion channels (*Kcnma1, Scn5a, Kcnq4*), precursor proteins (*Avp, Hcrt*). It was speculated that these genes were crucial for the pathogenesis of FS.

### Hyperthermia Induced Seizures could be Reduced by the DPP4 Inhibitor Sitagliptin

**Figure 4:**
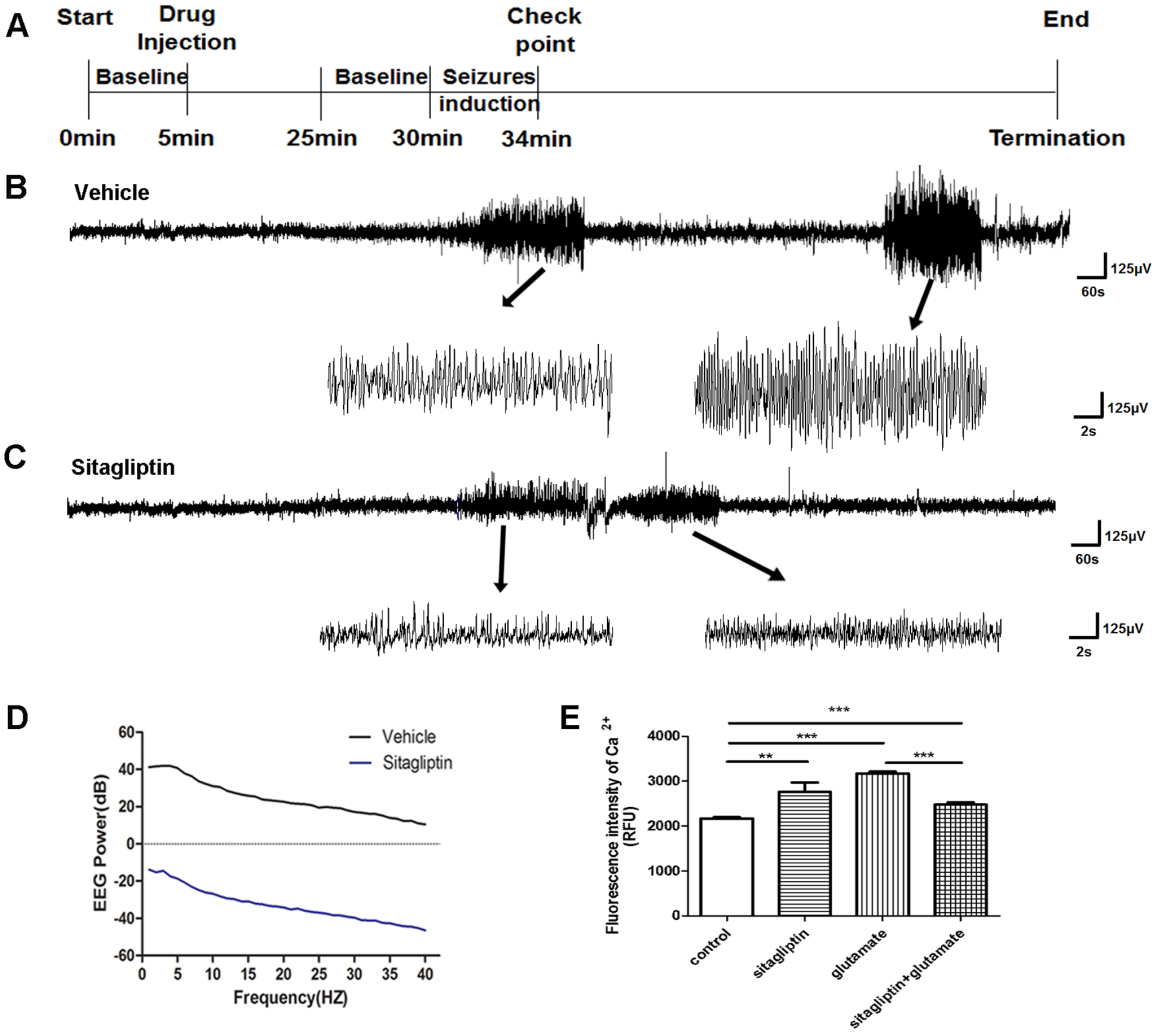
Anticonvulsant effects of sitagliptin in rats exposure to hyperthermia. (A) Schematic diagram of experimental protocol. Data were binned in 60 min intervals. (B) The representative EEG tracings from rats in vehicle group (n = 6). Hyperthermia-induced seizure were shown in details in the expanded lower trace during seizure induction period (left panel) or observation period (right panel). (C) The representative EEG tracings from rats in sitagliptin group (n = 6). Hyperthermia-induced seizure were shown in details in the expanded lower trace during seizure induction period (left panel) or observation period (right panel). (D) Power spectral analysis of EEG between the two groups. Differences between the vehicle (black line) and sitagliptin groups (blue line) were statistically significant (*P*<0.001). The vertical axis was the average EEG power spectral (dB), and the horizontal axis was the frequency (HZ). (E) C6 cells in control group were treated with 5 µl PBS; sitagliptin group were treated with 100 µM sitagliptin; glutamate group were treated with 60 µM glutamate; sitagliptin+glutamate group were treated with both 100 µM sitagliptin and 60 µM glutamate. **P<0.01, ***P<0.001.

DPP4 was chosen to explore the role of DEGs in FS pathogenesis. To study this, icv injection of sitagliptin was applied during EEG power analysis (Fig. 4A). Rats were exposed to hyperthermia at a core temperature of 44°C for 4 min after sitagliptin (2 mg/kg, n = 6) or vehicle (normal saline) was injected icv. The EEG results revealed that sitagliptin decreased both the frequency and the amplitude of epileptiform spikes significantly compared to that of the vehicle treated group. The average EEG power spectrum in the whole frequency band (0–40 Hz) was significantly lower (*P*<0.001, independent *t*-test) in the sitagliptin treated group compared to the vehicle treated group (Fig. 4B–D). These data suggest that DPP4 may plays a critical role in seizure generation, which can be attenuated by a DPP4 inhibitor.

The rat C6 glioma cell line was used to explore the function of sitagliptin *in vitro*. We hypothesized that the method by which sitagliptin protects neurons from hyperexcitability is by stabilizing cellular Ca^2+^ homeostasis. Intracellular Ca^2+^ (Fig. 4E) revealed that although sitagliptin caused a rapid increasing [Ca^2+^]i (2169±32.07 vs 2761±213.3, *P*<0.01), it significantly decreased glutamate induced [Ca^2+^]i increases (3167±50.44 vs 2475±52.14, *P*<0.001). These data suggests that the neuroprotective role of sitagliptin may occur via the stabilization of cellular Ca^2+^ homeostasis.

### Different DNA Methylation Rate of DPP4 between HP and HR Rats

**Figure 5:**
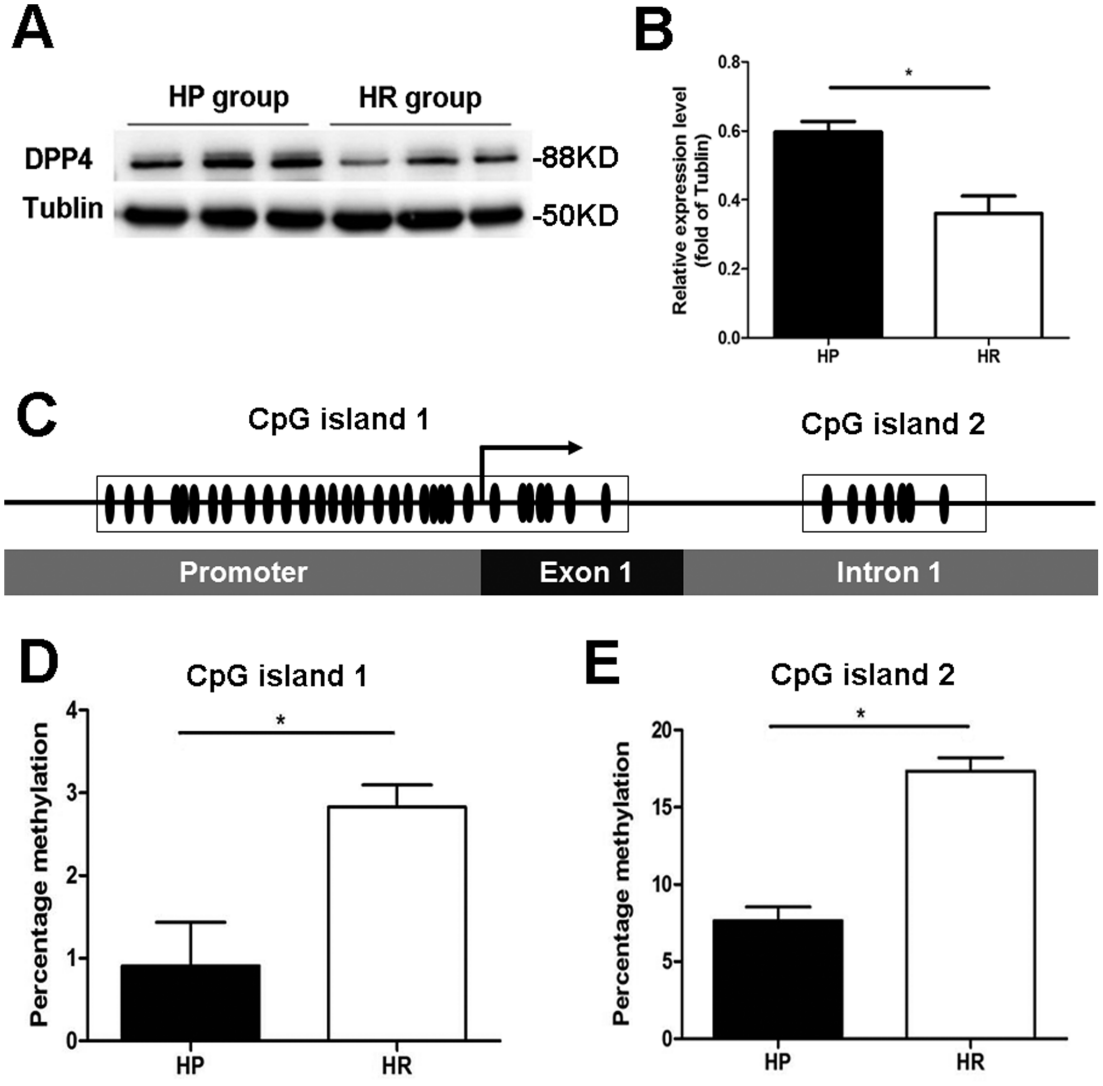
Expression and DNA methylation analysis of *DPP4*. (A) Different DPP4 expression in hippocampal of HP rats (n = 3) and HR rats (n = 3). (B) Gray Analysis data of DPP4 expression in hippocampal between HP rats and HR rats, data were normalized to the housekeeping gene Tublin, *P<0.05. (C) Sketch map about the location of CpG island 1 and CpG island 2 in DNA sequence of *DPP4,* black box show the rang of CpG island, elliptic point mean CG site. (D) HP rats (n = 3) show less methylated CG sites than HR rats (n = 3) in *DPP4* CpG island 1. (E) HP rats (n = 3) show less methylated CG sites than HR rats (n = 3) in DPP4 CpG island 2. The percentage methylation ratio were statistic from three independent experiments, *P<0.05.

It was observed that the *DPP4* mRNA expression was significantly greater (FC = 2.5367, *P*<0.05) in the HP group compared to the HR group (Fig. 1D), which is consistent with the protein productions of these two groups. Compared to HR rats, the hippocampus of HP rats showed a significant elevation (0.5961±0.0296 vs 0.3606±0.0500, *P*<0.05) in the expression of DPP4 (Fig. 5A–B). Meanwhile, immunohistochemical results from the hippocampus of HP rats revealed higher DPP4 expression than that observed in HR rats (Fig. S1). Subsequently, whether DNA methylation mediate the different expression levels of DPP4 between HP and HR rats was explored. Two CpG islands were analyzed from the DNA sequence of *DPP4:* CpG island 1 (−229/+94) and CpG island 2 (+221/+325). CpG island 1 contains 31 CG sites, 24 of which belong to the promoter and 7 of which belong to exon 1. All 7 CG sites of CpG island 2 belong to intron 1 (Fig. 5C). Bisulfite sequencing revealed a lower DNA methylation rate in HP rats than in HR rats in both CpG island 1 (0.9090±0.5248 vs 2.828±0.2672, *P*<0.05) and CpG island 2 (7.667±0.8819 vs 17.33±0.8819, *P*<0.01) of *DPP4* (Fig. 5D–E).

## Discussion

Gene expression profile analysis was used in this study to identify candidate susceptibility genes and expression patterns for FS in rat models. According to the results of the Hierarchical Cluster Analysis, all experimental samples were divided into two different clusters based on expression similarity, and we can conclude that these genetic results are consist with with the animals behavior (HP vs HR) [20], which provides a solid foundation for microarray analysis. Using a range of data mining and information annotation approaches, we were able to identify a number of transcriptome features that may shed light on the pathogenesis of FS.

We identified some important genes whose GO and signal pathways may be associated with the pathogenic factors of FS (Fig. 2A–B). Upregulated genes were associated with the cellular response to *IFN-γ* and *IL-6*. In addition, downregulated genes were associated with the negative regulation of T cell proliferation and immune system processes, which may help clarify the role of immunity and inflammation in FS. An increasing amount of evidence supports the involvement of immune and inflammatory processes in the etiopathogenesis of seizures [23], [24]. Various experimental observations provide evidence for an active role of brain-derived inflammatory molecules and the activation of related cell signaling pathways (e.g., the IL-1 receptor/Toll-like receptor pathway, NF-κB pathway and MAPK pathway) in seizure pathogenesis [25]. According to our KEGG pathway analysis, the steroid hormone biosynthesis pathway was the most significant pathways of upregulated genes (Fig. 2C), which concurs with research reporting that neurosteroids are synthesized within the brain and rapidly modulate neuronal excitability [26], [27]. However, an association between steroid hormones and the immune response in the brain has also been detected [28]. Because the inflammatory response could have serious detrimental effects in the brain, local steroid generation negatively regulates NF-κB dependent signaling pathways, and therefore serves as an important element for the control of inflammation in the central nervous system [28].

Using advanced molecular network techniques, we were able to propose a molecular network (Fig. 3). In this network, *IL-1β* and *IL-6* were located in the center of the constructed network map, because they might have a more important function than any of the other genes in the network. *IL-1β*, as a known susceptibility gene in FS, is responsible for enhancing susceptibility to hyperthermia-induced seizures in immature rats and increasing susceptibility to long-lasting seizures in adult rats [6], [29], [30]. The constitutively low level of *IL-6* mRNA increased immediately following a seizure [31], [32]. Consequently, IL-6 overexpression could increase seizure sensitivity by impairing GABAergic neurons in the hippocampus [33]. In addition, another differentially expressed gene, *DPP4 (CD26),* is directly related to *IL-6* in the constructed network map (Fig. 3), which suggests a potential role for DPP4 in FS. DPP4 is a T cell surface antigen and dipeptidyl peptidase that can hydrolyze various substrates, including gastrointestinal hormones (e.g., Glp-1 and Glp-2), neuropeptide-Y (NPY), chemokines and others [34]. Previous studies revealed that glucagon-like peptide 1 (Glp-1) acts directly through the Glp-1 receptor (GLP-1R) pathway to attenuate the effect of seizures [35]. Glp-1R deficient mice have enhanced seizure severity and neuronal excitability [36]. NPY was also known to be an important modulator of excitability in the central nervous system, and overexpression of NPY in the rat hippocampus could mediate anticonvulsant effect [37], [38]. Therefore, the up-regulation of DPP4 in the rat hippocampus may cause a reduction of its substrates, such as GLP-1 and NPY, and thereby reduced the seizure threshold of the animals.

In addition to gene network analysis, our work reveals that co-expression networks were likely to exhibit a high degree of functional redundancy in targeting similar sets of downstream genes (Table S5). Ion channels play critical roles in regulating neuronal excitability and contribute to epileptogenesis. Various types of ion channels are involved in seizures, particularly voltage-gated sodium channels and GABA_A_ receptors [39], [40]. We observed some ion channel genes (e.g., *potassium large conductance calcium-activated channel, subfamily M, alpha member 1 [Kcnma1]* and *sodium channel, voltage-gated, type V, alpha subunit [Scn5a]*) in our co-expression network, which provides evidence that can lead to a better understanding of the role of ion channels in FS. Kcnma1, also known as the BK**_Ca_** channel, is activated by both elevated [Ca^2+^]i and membrane depolarization. Studies have shown that presynaptic BK**_Ca_** channels are recruited only following a massive [Ca^2+^]i accumulation in the presynaptic terminals, such as during seizures, which can inhibit depolarization-induced bursting activity by hyperpolarizing the presynaptic membrane, which can then attenuate neuronal hyperexcitability and oppose neuronal activity [41], [42]. Downregulation of Kcnma1 expression in the HP group may be a potential pathway that can contribute to the occurrence of FS.

*DPP4 (CD26)* was selected to further explore the relationship between DEGs and FS. *DPP4* is located in FEB 3B and has scarcely been studied in relation to FS. As discussed previously, DPP4 is a member of the dipeptidyl peptidase family and a T cell surface antigen whose expression correlates with IL-1β and IL-6 expression in many organs and tissues, including the brain [43], [44]. To confirm whether DPP4 may affect seizure generation, we performed EEG power analysis using sitagliptin, which is a DPP4 inhibitor that has a neuroprotective function [45]. Sitagliptin rapidly decreased the frequency and amplitude of epileptiform spikes induced by hyperthermia (Fig. 4). In our study, we found that the DPP4 inhibitor sitagliptin could prevent the glutamate induced rapid increase in [Ca^2+^]I, while sitagliptin itself can increase [Ca^2+^]i as well, which suggests that sitagliptin may play a role in stabilizing cellular calcium homeostasis and protecting neurons against excitotoxicity. In addition, it has been reported that GLP-1 exposure caused a rapid increase in [Ca^2+^]i in hippocampal neurons and prevented glutamate induced Ca^2+^ influx through voltage-dependent calcium channels [46]. Considering that GLP-1 is one of the substrate that is cleaved by DPP4, sitagliptin might exert its influence on Ca^2+^ regulation through a GLP-1 dependent mechanism. However, the mechanism behind the ability of GLP-1 to increase [Ca^2+^]i while decreasing glutamate induced Ca^2+^ influx is still unknown and should be further explored.

The two new strains of FS animal models were established by hyperthermia exposure, and epigenetic research has found that environmental factors such as temperature might mediate physiological or pathological gene expression by influencing DNA methylation and chromatin modifications [47]. We suggest that epigenetic modifications may play a role in developing the two kinds phenotypes through the regulation of gene expression, as evidenced by DPP4 expression and its DNA methylation changes. The *DPP4* gene locus contains two CpG islands in the promoter region. Hypermethylation of the *DPP4* promoter CpG island has been associated with the repression of gene expression and disease severity in human melanoma cell lines, T-cell leukemia and obese[48]–[50]. Therefore, we analyzed the CpG islands of *DPP4* in the hippocampus between the two groups. A significantly lower DNA methylation rate was found in HP rats compared to HR rats at both CpG island 1 and CpG island 2 (Fig. 5D–E). Therefore, DNA methylation may have a substantial impact on DPP4 gene expression in hippocampus. The differential DNA methylation may occur for two possible reasons. The first possibility is the difference in seizure sensitivity. Rapid H3 and H4 modification after seizures has been observed, and these modifications correlate with DNA methylation and mRNA alteration [51], [52]. The second possible cause is the different temperatures in the model processing (41°C for HP rats and 43°C for HR rats). It has been reported that dynamic chromatin modification plays a critical role in postnatal thermotolerance acquisition [53]. Previous research also indicates that there exist postnatal epigenetic influences on seizure susceptibility in a kindling model [13]. Our research would provides further evidence to support a role for epigenetic mechanisms in FS pathogenesis.

In summary, based on the above results, we have analyzed our microarray data intensively and identified a number of DEGs and expression patterns between the HP and HR groups. Moreover, our microarray results provide important evidences that inflammation involved in FS. The study of DPP4 confirmed that DEGs verified by our microarray may associated with FS. *DPP4* gene DNA methylation analysis provide a possibility that epigenetic mechanism involved in FS, which need to be further determined. However, there are still a lot of information within the dataset that remains to be elucidated, and which may provide more insights into the pathogenesis of FS. We hope to provide useful information for elucidating the responsible pathogenic factors of FS, for discovering biomarkers for epileptogenesis and for defining windows of opportunity for therapeutic and/or preventive interventions.

## Supporting Information

Figure S1 Immunohistochemistry detection of DPP4 in hippocampal samples. The representative DPP4 expression of HP rats (n = 3) in hippocampal (A), CA3 (C), and dentate gyrus (E), and representative DPP4 expression of HR rats (n = 3) in hippocampal (B), CA3 (D), and dentate gyrus (F).
(TIF)

Table S1 The Differential Expressed Genes between the HP group and the HR group.
(XLS)

Table S2 Sequences of Primers for qRT-PCR.
(XLS)

Table S3 Significant GO analysis between the HP group and the HR group.
(XLS)

Table S4 Significant Pathways analysis between the HP group and the HR group.
(XLS)

Table S5 38 genes identified by gene co-expression network.
(XLS)
