# Supplementary figures and images for: Transcriptome Analysis of the Hippocampus in Novel Rat Model of Febrile Seizures
Source: PLoS One. 2014 Apr 15;9(4):e95237. doi: 10.1371/journal.pone.0095237 (PMC3988142; doi:10.1371/journal.pone.0095237)

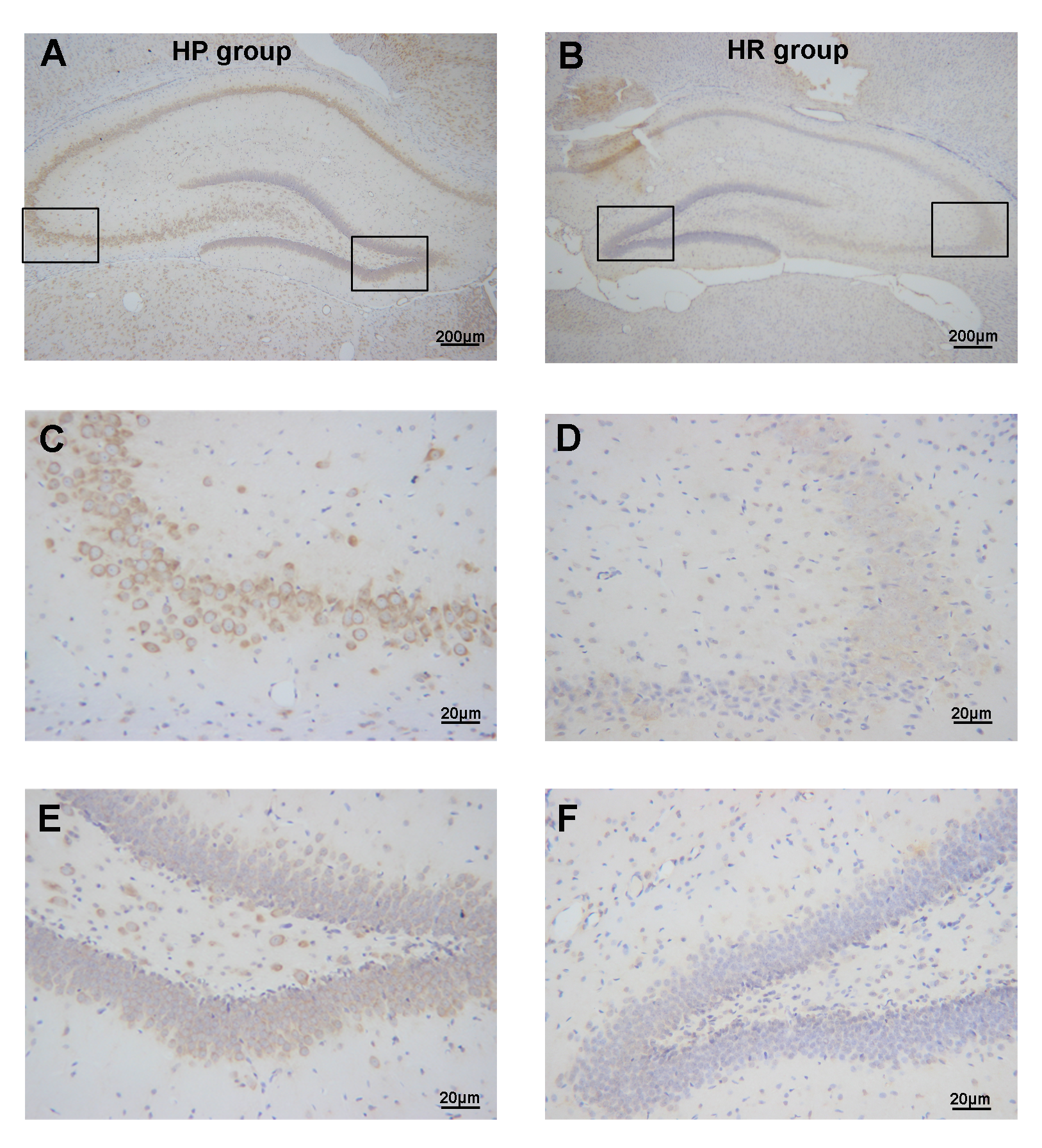

Supplement: Figure S1 — Immunohistochemistry detection of DPP4 in hippocampal samples. The representative DPP4 expression of HP rats (n = 3) in hippocampal (A), CA3 (C), and dentate gyrus (E), and representative DPP4 expression of HR rats (n = 3) in hippocampal (B), CA3 (D), and dentate gyrus (F). (TIF) [file pone.0095237.s001.tif]
